# RIPK1 S213E mutant suppresses RIPK1-dependent cell death by preventing interactions with RIPK3 and CASP8

**DOI:** 10.1038/s41420-025-02647-x

**Published:** 2025-07-25

**Authors:** Ning Nan, Hong Hu, Xinxin Zhu, Jia Liu, Feiyang Yuan, Zhijie Li, Huayi Wang

**Affiliations:** 1 School of Life Science and Technology, ShanghaiTech University, Shanghai, China; 2 CAS Center for Excellence in Molecular Cell Science, Shanghai Institute of Biochemistry and Cell Biology, Chinese Academy of Sciences, University of Chinese Academy of Sciences, Shanghai, China; 3 University of Chinese Academy of Sciences, Beijing, China; 4 Beigene, Ltd., Shanghai, China

**Keywords:** Necroptosis, Apoptosis

## Abstract

RIPK1 (Receptor-interacting serine/threonine-protein kinase 1) is fundamental in regulating cell proliferation, programmed cell death, and inflammation. Within the TNF (tumor necrosis factor) signaling pathway, the kinase activity of RIPK1 is essential for determining cellular fate, promoting either apoptosis or necroptosis. Mutations disrupting RIPK1 kinase activity significantly impact cellular fate decisions, highlighting its importance in the TNF signaling cascade. This study generated and characterized a novel mutation of human RIPK1, S213E that exhibits unique inhibitory properties. Although located in the kinase domain, the S213E mutation disrupts RIPK1 homodimerization and its interactions with downstream effectors, such as RIPK3, without directly suppressing RIPK1 kinase activity. These findings indicate that the S213E mutation converts RIPK1 into a super-autoinhibitory state, effectively isolating it from downstream effectors involved in both apoptosis and necroptosis.

## Introduction

RIPK1 is a pivotal upstream regulator that governs cell proliferation, death, and inflammation [1]. Structurally, it comprises an N-terminal kinase domain (KD), a C-terminal death domain (DD), and an intermediate domain featuring the RIP homotypic interaction motif (RHIM). Extensive research has been conducted on the multifaceted functions of RIPK1 within the tumor necrosis factor receptor 1 (TNFR1) signaling pathways [2]. Upon TNFα stimulation, TNFR1 rapidly recruits TRADD, which in turn attracts RIPK1 along with E3 ubiquitin ligases cIAP1/2 and TRAF2/5 to form the TNFR1 signaling complex (TNF-RSC, alternatively known as Complex I) [3–7]. This complex plays a crucial role in NF-κB activation, promoting RIPK1-mediated cell proliferation and inflammation [3–5, 7–9]. When complex I is disrupted by inhibiting either NF-κB or cIAP1/2, RIPK1-mediated apoptosis and necroptosis become activated. This process involves the recruitment of Fas-associated death domain protein (FADD) and caspase-8 (CASP8) to RIPK1, leading to the formation of Complex IIa to activate apoptosis [10, 11]. While upon CASP8 inhibition, RIPK1 will recruit receptor-interacting serine/threonine-protein kinase 3 (RIPK3) and the downstream pseudokinase mixed lineage kinase domain-like (MLKL) to form Complex IIb. This transition from Complex IIa to Complex IIb (necrosome) ultimately results in necroptosis [12–15].

The kinase activity of RIPK1 has been established as crucial for RIPK1-mediated cell death. Consequently, kinase-dead mutants or RIPK1 kinase inhibitors, such as Necrostatin-1 (NEC-1), can effectively block the transmission of cell death signals [1, 9]. RIPK1 undergoes extensive post-translational modifications, including phosphorylation and ubiquitylation, which regulate its function during TNF signaling [16]. Upon activation, RIPK1 undergoes autophosphorylation at sites such as S161 and S166, with S166 phosphorylation serving as a marker of activation [17]. Other kinases, like IKKα/β, TBK1 and MK2, phosphorylate RIPK1 at S25, T189, S320 and S336, respectively, inducing conformational changes that inhibit kinase activation and prevent cell death [18–22]. Additionally, RIPK1 homodimerization through the “RHIM” motif and death domain is crucial for RIPK1-mediated cell death, and ubiquitylation at K627 disrupts homodimerization and inhibits cell death [8].

This study characterizes a novel S213E mutation in human RIPK1 that blocks RIPK1 autophosphorylation at S166 and effectively inhibits TNFα-induced apoptosis and necroptosis in HT-29 cells. The defect in RIPK1 autophosphorylation in this mutant is not due to kinase activity inhibition but rather due to its inability to get phosphorylated. Mechanistic investigations reveal that the S213E mutant of RIPK1 blocks its homodimerization, a crucial step in both apoptosis and necroptosis signaling pathways. Additionally, this mutant disrupts the direct interactions between RIPK1 and CASP8, as well as between RIPK1 and RIPK3, thereby effectively inhibiting RIPK1-dependent apoptosis and necroptosis, respectively. Our research reveals that the S213E mutation in human RIPK1 induces a super-autoinhibitory state, disrupting homodimerization and interactions with RIPK3 or CASP8, thereby preventing RIPK1-mediated cell death. This discovery highlights the potential therapeutic benefits of targeting the super autoinhibitory conformation in diseases associated with RIPK1-mediated cell death.

## Results

### S213E mutant blocks RIPK1 autophosphorylation and necroptosis

**Fig. 1:**
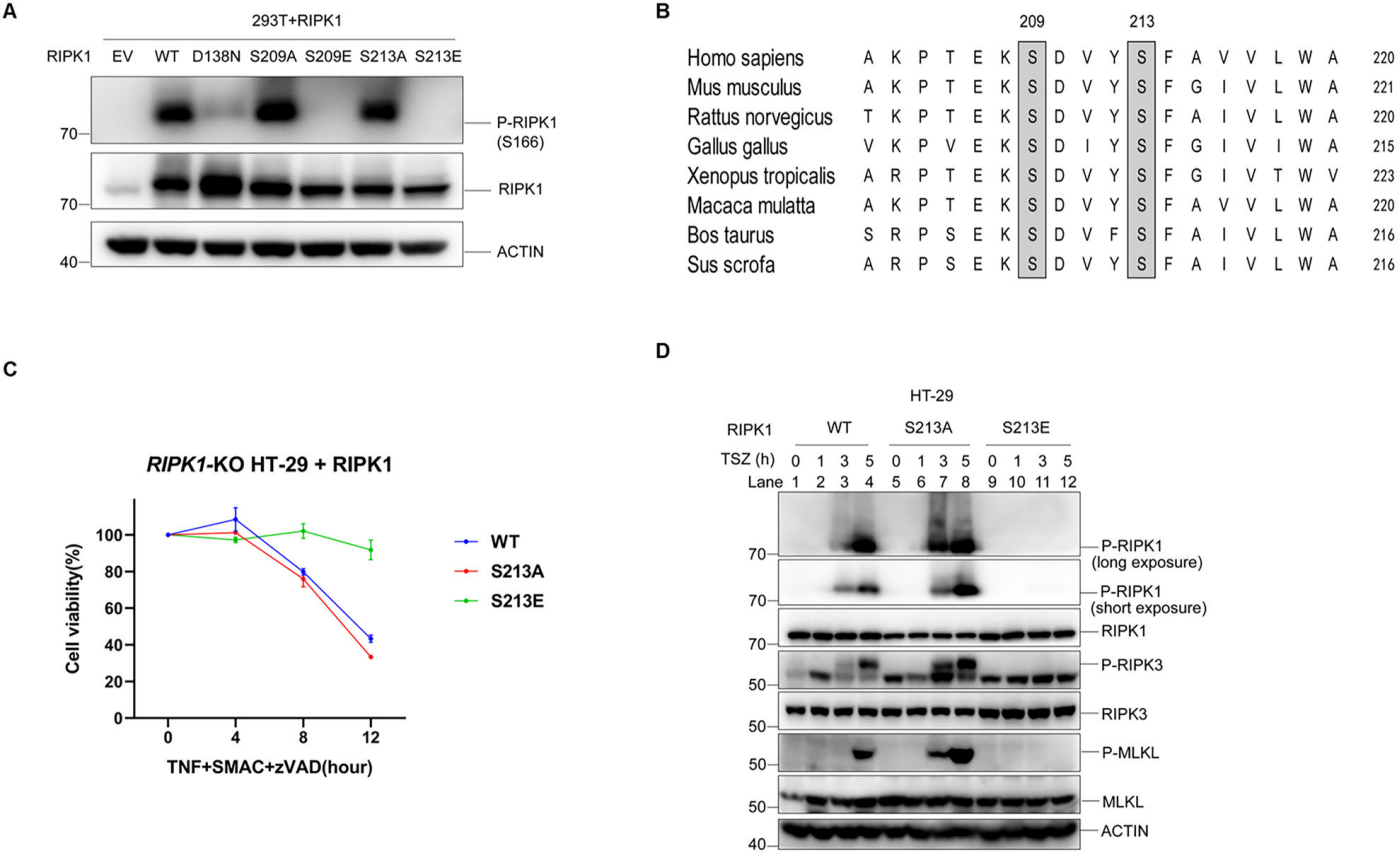
S213E mutant blocks RIPK1 autophosphorylation and necroptosis. **A** HEK293T cells overexpressed wild-type (WT) or indicated RIPK1 mutants, the levels of RIPK1 S166 autophosphorylation was determined by immunoblotting using anti-P-RIPK1 (S166) antibody. EV: empty vector. **B** Sequence alignment of conserved serine in the RIPK1 orthologs across different species. The conserved sequences flanking S209 and S213 of human RIPK1 were used as query sequences in a gapped BLAST search. The conserved serine residues were squared in gray. **C** HT-29 *RIPK1*-KO cells were transfected with lentiviral vectors encoding wild-type or indicated RIPK1 mutants. Necroptosis was induced by treating with TNF + SMAC + zVAD for indicated period of times, cell viability was measured by CellTiter-Glo assay. **D** HT-29 cells of indicated RIPK1 genotypes were treated with TSZ for indicated time points, the levels of P-RIPK1 (S166), P-RIPK3 (S227), P-MLKL (S358) signal were determined by immunoblotting using antibodies as indicated. Short exposure: 10 s; long exposure: 1 min. If not stated specifically in this study, human TNFα (T or TNF): 20 ng/ml; Smac mimetic (S or SMAC): 100 nM; zVAD.fmk (Z or zVAD): 20 μM; Necrostatin-1 (NEC-1): 10 μM. The data are represented as the mean ± SD of *n* = 3 independent experiments.

To discover new functional phosphorylation sites in the kinase domain of RIPK1, we individually mutated all serine (S) and threonine (T) residues to glutamate (E), generating a phospho-mimic mutation library. After transfecting the mutated RIPK1 genes into HEK293T cells, we screened for autophosphorylation at the S166 site to identify mutations that affected kinase activity. Notably, we discovered that the phospho-mimic mutations on S209 and S213 that were conserved in the RIPK1 sequence across different species significantly inhibited autophosphorylation at S166 (Fig. 1A, B). To study how the phospho-mimic mutations S209E and S213E affect necroptosis, we used human colon cancer cells (HT-29) that lack RIPK1 (RIPK1-KO). Our research shows that cells with the S213E mutation are unable to undergo necroptosis, whereas cells with the S209E mutation show slightly reduced necroptosis. On the other hand, cells carrying the phospho-dead mutant S213A mutation exhibited a slight increase in necroptosis compared to normal cells (Fig. 1C, Supplementary Fig. 1A). This observation is supported by the finding that the levels of autophosphorylation of RIPK1 and RIPK3, as well as the phosphorylation of MLKL, were significantly higher in cells expressing RIPK1-S213A compared to those expressing wild-type RIPK1. In contrast, no detectable phosphorylation occurred in cells expressing the S213E mutation (Fig. 1D). Similar results were obtained with another phospho-mimic mutant (S213D) and a phospho-dead mutant (S213C), which mirrored the findings with S213E and S213A respectively (Supplementary Fig. 1B). These results indicate that S213 could potentially serve as a crucial phosphorylation site that functions as a negative regulator of RIPK1 activity. To evaluate whether the S213E mutation has a functional role beyond TNF-induced necroptosis signaling, additional necroptosis-inducing cytokines such as IFN-γ, TRAIL, Poly I:C, and LPS were used to test the function of S213E [2, 23]. The results demonstrated that S213E was able to block necroptosis induced by all these cytokines (Supplementary Fig. 1C–F). This suggests that the S213E mutation has a broader functional role in regulating necroptosis, not limited to TNF-induced signaling. However, phosphorylation at S213 has not been detected in any previous small or large-scale studies.

### S213E does not inhibit the kinase activity of RIPK1

**Fig. 2:**
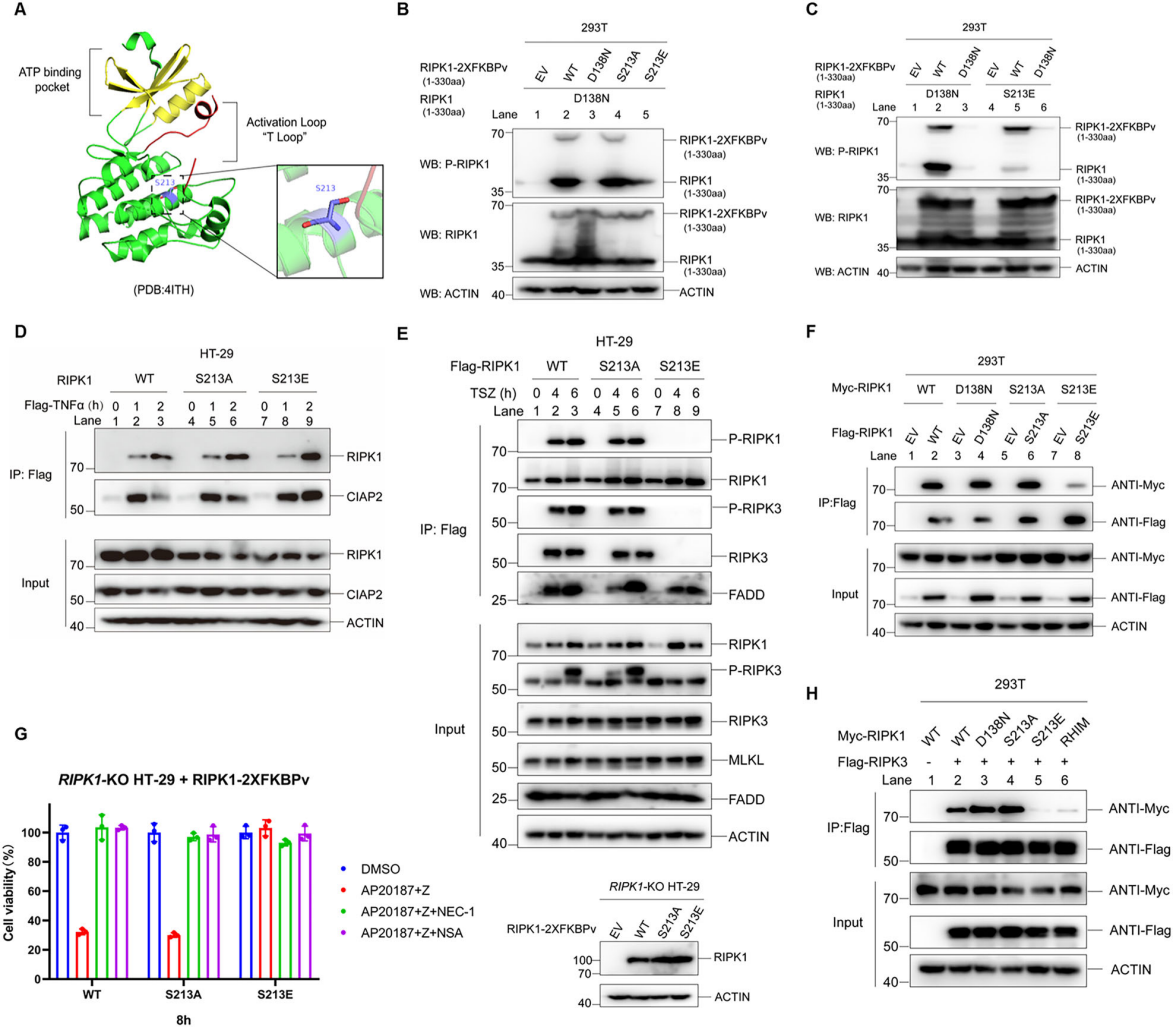
S213E disrupts RIPK1 homodimerization and the interaction between RIPK1 and RIPK3. **A** The structural of the RIPK1 kinase domain (PDB: 4ITH). The ATP binding pocket and Activation Loop “T loop” are shown in yellow and red, respectively, while S213 site is drawn in purple; the side chain of S213 site is highlighted and displayed in the stick-ball model. HEK293T cells were co-transfected with expression vectors for RIPK1 (1–330aa)-D138N and RIPK1 (1–330aa)-WT-2XFKBPv, RIPK1 (1–330aa)-D138N-2XFKBPv, RIPK1 (1–330aa)-S213A-2XFKBPv, RIPK1 (1-330aa)-S213E-2XFKBPv (**B**) or that of RIPK1 (1-330aa)-D138N, RIPK1 (1–330aa)-S213E and RIPK1 (1–330aa)-WT-2XFKBPv, RIPK1 (1–330aa)-D138N-2XFKBPv (**C**) as indicated for 18 h. The levels of P-RIPK1 (S166) were determined by immunoblotting using anti-P-RIPK1 (S166) antibody. EV empty vector. **D** HT-29 cells of indicated RIPK1 genotypes were stimulated with Flag-TNFα for indicated time points. The cell lysates were immunoprecipitated with anti-Flag beads. The recruitment of RIPK1 was analyzed by immunoblotting. Flag-TNFα: Flag tag fused mTNFα (10 μg/ml). CIAP2 was a control for Complex I. **E** HT-29 *RIPK1*-KO cells stably expressing Flag-RIPK1 of indicated genotypes were stimulated with TSZ for indicated time points. The cell lysates were immunoprecipitated using anti-Flag beads. The recruitment of RIPK3 and P-RIPK3 (S227) was analyzed by immunoblotting using antibodies as indicated. **F** HEK293T cells were co-transfected with expression vectors for Flag-RIPK1-WT, Flag-RIPK1-D138N, Flag-RIPK1-S213A, Flag-RIPK1-S213E and Myc-RIPK1-WT, Myc-RIPK1-D138N, Myc-RIPK1-S213A, Myc-RIPK1-S213E as indicated for 24 h. The cell lysates were immunoprecipitated using anti-Flag beads. The protein intereaction was analyzed by immunoblotting using antibodies as indicated. EV: empty vector. **G** HT-29 *RIPK1*-KO cells stably expressing RIPK1-2XFKBPv of indicated genotypes were treated with AP20187+zVAD in the presence or absence of NEC-1 or NSA for 8 h, cell viability was measured by CellTiter-Glo assay (**G. left**). The expression of RIPK1-2XFKBPv mutants were determined by immunoblotting (**G. right**). AP20187: 500 nM; NSA: 2 μM. **H** HEK293T cells were co-transfected with expression vectors for Flag-RIPK3 and Myc-RIPK1-WT, Myc-RIPK1-D138N, Myc-RIPK1-S213A, Myc-RIPK1-S213E, Myc-RIPK1-RHIM as indicated for 24 h. The cell lysates were immunoprecipitated using ANTI-Flag beads. The protein intereaction was analyzed by immunoblotting using antibodies as indicated. RHIM: RIPK1 RHIM motif core sequences “IQIG” mutant to “AAAA”. The data are represented as the mean ± SD of *n* = 3 independent experiments.

The S213E mutation could fully inhibit TNF-induced RIPK1 autophosphorylation at S166, hinting that it may directly suppress RIPK1’s kinase activity. However, the crystal structure data of the RIPK1 kinase domain reveals that S213 is not situated within the “ATP binding pocket” or “T loop” regions, indicating that the S213E mutation unlikely impacts the kinase activity (Fig. 2A). To investigate how the S213E mutation influences autophosphorylation at S166, we conducted biochemical experiments by co-expressing plasmids encoding both the RIPK1 kinase domain (KD) and a RIPK1 KD fused with a chemical-induced dimerization domain (2x FKBPv) in HEK293T cells. The results indicate that the S213E mutant does not undergo autophosphorylation at S166 but retains the capacity to phosphorylate the D138N mutant, albeit at lower levels compared to the wild-type (WT). Specifically, by comparing lanes 2 and 3 to lanes 4 and 5 in Fig. 2B, it is clear that the S213E mutant can phosphorylate D138N but not itself. Furthermore, the S213E mutant cannot be phosphorylated by the WT RIPK1, as evidenced by comparing lane 2 with lane 5 in Fig. 2C. Additional in vitro kinase assays utilizing purified wild-type or RIPK1 kinase domain (KD) mutant proteins to phosphorylate GST-RIPK1 KD-D138N proteins further validate these findings, showing that S213E can still phosphorylate D138N on S166 without undergoing autophosphorylation (comparing lanes 2 and 3 with lanes 4 and 5 in Supplementary Fig. 2A). These data indicate that, unlike the kinase-dead mutation D138N, the kinase activity of S213E is partially retained, while it nearly loses its function as a substrate for RIPK1 autophosphorylation. This may be due to conformational changes in RIPK1 caused by the S213E mutation.

### S213E disrupts RIPK1 homodimerization and the interaction between RIPK1 and RIPK3

Considering that the kinase activity of RIPK1, and not autophosphorylation at S166, is crucial for RIPK1-mediated necroptosis [24]. The retention of kinase activity by the S213E mutant suggests other functional deficiencies in S213E-expressing cells may be responsible for inhibiting necroptosis. In TNF signaling, TNFR1 signaling complex I and the necroptosis complex (Complex IIb or necrosome) undergo sequential activation, with RIPK1 serving as a critical component for the proper functioning of both [25]. Our findings indicate that the S213E mutation does not hinder RIPK1’s ability to form Complex I, as demonstrated by immunoprecipitation of Flag-TNF in HT-29 cells (Fig. 2D). Consistently, the activations of NF-κB or MAPK pathway-related proteins in S213E cells showed no significant differences compared to those in WT cells (Supplementary Fig. 2B). These results suggest that the S213E mutation does not affect RIPK1-induced activations of either the NF-κB or MAPK pathways. However, in cells expressing the S213E mutant, the formation of the necrosome was disrupted, as evidenced by the absence of binding between RIPK1 and RIPK3 observed through Flag-RIPK1 immunoprecipitation upon necroptosis induction (Fig. 2E). Interestingly, the S213E mutation did not prevent RIPK1 assembly into Complex II, as evidenced by the unchanged TNF-induced binding between RIPK1 (S213E) and FADD (Fig. 2E). This indicates that the transition from Complex I to Complex II occurred successfully. Furthermore, the formation of Complex II is triggered by RIPK1 homodimerization via self-interaction within the RHIM or death domain (DD). This not only boosts RIPK1’s kinase activity but also enables it to interact more effectively with other proteins that possess either the RHIM or death domain [26]. Subsequently, the impact of the S213E mutation on RIPK1 homodimerization was evaluated through Co-IP assays in HEK293T cells, revealing a significant reduction in RIPK1 homodimerization (Fig. 2F). Additionally, when the core sequences “IQIG” in the RHIM were mutated to “AAAA” or the death domain (DD) was truncated, neither RIPK1-4A-S213E nor RIPK1-ΔDD-S213E could bind to full-length RIPK1-S213E (Supplementary Fig. 2C). These observations indicate that, despite the S213 site being positioned within the kinase domain (KD), the S213E mutation disrupts RIPK1 homodimerization facilitated by both the RHIM and DD. Previous studies have also reported the disruption of RIPK1 homodimerization by multiple mutations within the DD, including K599R, R603Q, and K627R, which subsequently block necroptosis [8, 27, 28]. Notably, the loss of function mutation in the DD can be rescued through chemical-induced dimerization by fusing 2× FKBPv at the C-terminus of RIPK1 [8, 27]. However, unlike these mutations in the DD, the chemical-induced dimerization of FKBPv failed to restore the function of RIPK1 (S213E) in necroptosis signaling (Fig. 2G). This indicates that the inhibitory function of S213E is not solely due to suppressing RIPK1 dimerization. The S213E mutation disrupts RIPK1 self-interaction facilitated by both the RHIM and DD (Fig. 2F). We further tested whether S213E could also directly disrupt the interaction between RIPK1 and RIPK3 through the RHIM. Our results demonstrated that the interaction between RIPK1-S213E and RIPK3 was drastically reduced, similar to the effect observed with a mutation in the core sequences of RHIM from “IQIG” to “AAAA” (Fig. 2H) [29]. Consequently, we conclude that the inhibition of necroptosis in S213E cells is attributable not only to the disruption of RIPK1 homodimerization but also to the interference with the interaction between RIPK1 and RIPK3, ultimately preventing necrosome formation.

### S213E mutant blocks RIPK1-dependent apoptosis

**Fig. 3:**
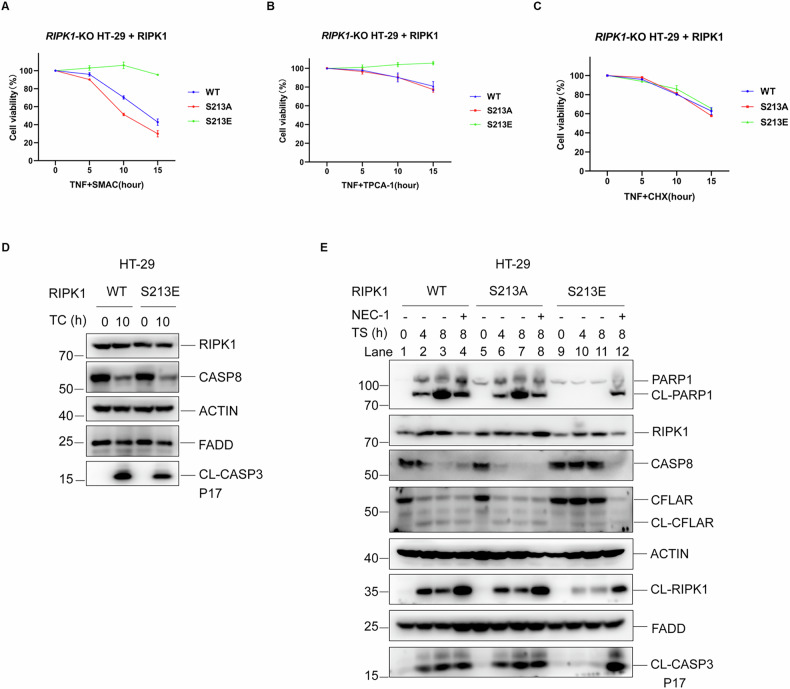
S213E mutant blocks RIPK1-dependent apoptosis. HT-29 cells of indicated RIPK1 genotypes were treated with TNF + SMAC (**A**), TNF + TPCA-1 (**B**) or CHX (**C**) for indicated period of times to induce apoptosis, cell viability was measured by CellTiter-Glo assay. TPCA-1: 20 μM. Cycloheximide (C or CHX): 2 μg/ml. **D** HT-29 cells of RIPK1 WT and S213E were treated with TC for indicated period of times. The levels of CASP8 and CL-CASP3 were determined by immunoblotting using antibodies as indicated. CL-CASP3: cleaved CASP3. **E** HT-29 cells of indicated RIPK1 genotypes were treated with TS in the presence or absence of NEC-1 for indicated period of times. The levels of CASP8, CFLAR, CL-CFLAR, CL-PARP1, CL-RIPK1, CL-CASP3 were determined by immunoblotting using antibodies as indicated. CL-CFLAR: cleaved CFLAR; CL-PARP1: cleaved PARP1; CL-RIPK1: cleaved RIPK1; CL-CASP3: cleaved CASP3. The data are represented as the mean ± SD of *n* = 3 independent experiments.

Previous research has demonstrated that the K599R mutation in RIPK1 disrupts homodimerization, potentially inhibiting both necroptosis and RIPK1-dependent apoptosis [27]. Similarly, the S213E mutation will likely block RIPK1-dependent apoptosis due to its ability to disrupt RIPK1 homodimerization. TNFR activation-induced apoptosis can be primarily categorized into RIPK1-dependent and RIPK1-independent types, which can be differentiated using various apoptosis sensitizers [30]. TNF can induce RIPK1-dependent apoptosis when combined with either SMAC mimics or NF-κB inhibitors, whereas it induces RIPK1-independent apoptosis when combined with cycloheximide (CHX) [1, 30–32]. In an experimental study utilizing HT-29 cells, the impact of mutations at the S213 position on both RIPK1-dependent and RIPK1-independent apoptosis were examined. The findings revealed a slight elevation in RIPK1-dependent apoptosis in cells with the S213A mutation, whereas the S213E mutation almost completely suppressed this apoptotic pathway (Fig. 3A, B). In contrast, neither the S213A nor the S213E mutation exhibited any influence on RIPK1-independent apoptosis (Fig. 3C, D). Consistent with this finding, caspase activity was inhibited in S213E cells, as demonstrated by the prevention of cleavage of RIPK1, CFLAR, CASP3, and PARP1 upon stimulation with TNF and SMAC mimics, as shown in Fig. 3E. Similar to necroptosis, we investigated whether the S213E mutant could prevent RIPK1-mediated apoptosis triggered by other cytokines. The results showed that the S213E mutant also inhibited RIPK1-dependent apoptosis induced by IFN-γ, TRAIL, Poly I:C, and LPS (Supplementary Fig. 3A–D). This observation implies that the S213E mutation alters the conformation of the RIPK1 kinase domain, resulting in allosteric effects that impair the mutant protein’s ability to induce apoptosis.

### S213E mutant does not disrupt the formation of Complex IIa, but the interaction of RIPK1 and CASP8

**Fig. 4:**
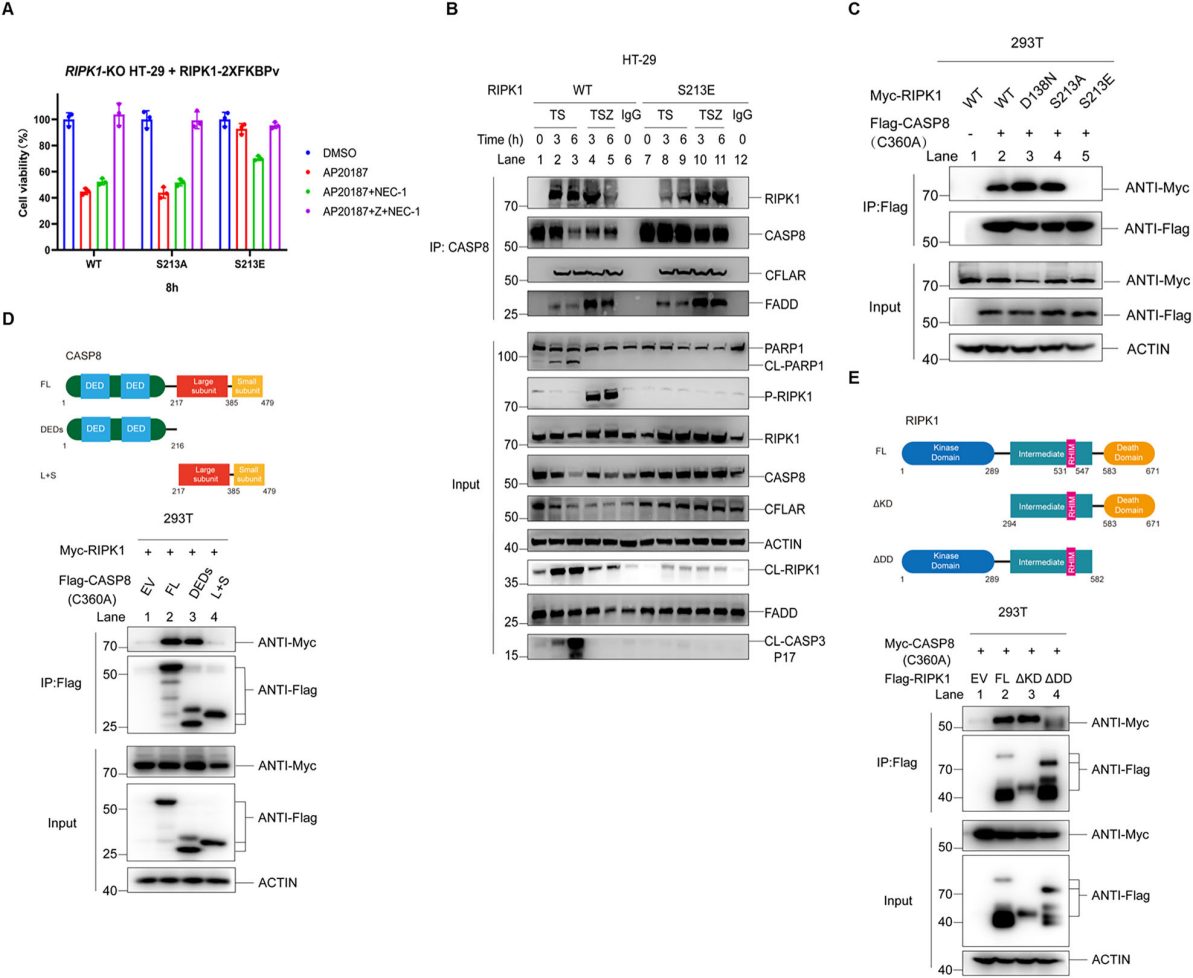
S213E mutant does not disrupt the formation of Complex IIa, but the interaction of RIPK1 and CASP8. **A** HT-29 *RIPK1*-KO cells stably expressing RIPK1-2XFKBPv of indicated genotypes were treated with AP20187, AP20187+zVAD in the presence or absence of NEC-1 for 8 h, cell viability was measured by CellTiter-Glo assay. AP20187: 500 nM. **B** HT-29 cells of indicated RIPK1 genotypes were stimulated with TS, TSZ for indicated time points. The cell lysates were immunoprecipitated with anti-CASP8 antibody. The recruitment of RIPK1, CFLAR, FADD and P-RIPK1 (S166), CL-PARP1, CL-RIPK1, CL-CFLAR, CL-CASP3 were analyzed by immunoblotting using antibodies as indicated. CL-CFLAR: cleaved CFLAR; CL-PARP1: cleaved PARP1; CL-RIPK1: cleaved RIPK1; CL-CASP3: cleaved CASP3. **C** HEK293T cells were co-transfected with expression vectors for Myc-RIPK1-WT, Myc-RIPK1-D138N, Myc-RIPK1-S213A, Myc-RIPK1-S213E and Flag-CASP8-C360A as indicated for 24 h. The cell lysates were immunoprecipitated using anti-Flag beads. The protein intereaction was analyzed by immunoblotting using antibodies as indicated. **D** A schematic diagram of CASP8 domain truncations (**D. top**). HEK293T cells were co-transfected with expression vectors for Flag-CASP8-C360A-FL, Flag-CASP8-C360A-DEDs, Flag-CASP8-C360A-L + S and Myc-RIPK1 as indicated for 24 h. The cell lysates were immunoprecipitated using anti-Flag beads. The protein intereaction was analyzed by immunoblotting using antibodies as indicated (**D. bottom**). EV: empty vector; FL: full length; DEDs: CASP8 truncation containing DED domains; L + S: CASP8 truncation containing large and small subunits. **E** A schematic diagram of RIPK1 domain truncations (**E. top**). HEK293T cells were co-transfected with expression vectors for Flag-RIPK1-FL, Flag-RIPK1-ΔKD, Flag-RIPK1-ΔDD and Myc-CASP8-C360A as indicated for 24 h. The cell lysates were immunoprecipitated using anti-Flag beads. The protein intereaction was analyzed by immunoblotting using antibodies as indicated (**E. bottom**). ΔKD, RIPK1 truncation without kinase Domain; ΔDD, RIPK1 truncation without Death Domain. The data are represented as the mean ± SD of *n* = 3 independent experiments.

The S213E mutant suppressed RIPK1-dependent apoptosis and necroptosis, much like the K599R mutant, which disrupts RIPK1 homodimerization. It has been reported that AP20187-induced dimerization of RIPK1 fused with 2x FKBPv can restore the ability of RIPK1(K599R) to induce RIPK1-dependent apoptosis and necroptosis [27]. However, when AP20187-induced dimerization was attempted on the S213E mutant, it failed to regain its ability to induce necroptosis and RIPK1-dependent apoptosis (Figs. 2G and 4A). This indicates that the S213E mutant’s inability to induce necroptosis and RIPK1-dependent apoptosis is not solely due to its blocking of RIPK1 homodimerization. It has been revealed that S213E can block the binding of RIPK3 to mutant RIPK1, thereby preventing the formation of the necrosome (Complex IIb) and inhibiting necroptosis (Fig. 2H). Subsequently, an analysis was performed to assess the effect of the S213E mutation on Complex IIa, the apoptosis signal complex. The results indicated that Complex IIa assembles properly in cells expressing the mutant S213E form of RIPK1. A comparison between wild-type RIPK1 and the S213E mutant revealed no significant defects in the components of Complex IIa, such as CASP8, CFLAR, and FADD (Fig. 4B). Notably, a slight decrease in the quantity of RIPK1 co-precipitating with CASP8 was observed in the CASP8-immunoprecipitates obtained from S213E cells (Fig. 4B, compare lanes 2 and 3 with lanes 8 and 9). These observations indicate a subtle alteration in the recruitment of RIPK1 to CASP8 in the mutant cells, which does not appear to disrupt the overall assembly of Complex IIa. However, this alteration significantly reduces CASP8 activity, as evidenced by the suppression of cleavage of CASP8 substrates such as RIPK1, CASP3, and PARP1 (Fig. 4B). Previous studies have suggested that RIPK1 recruits CASP8 through FADD in the context of RIPK1-dependent apoptosis [1, 2, 10, 30, 33]. The removal of FADD has been shown to suppress apoptosis but instead induce necroptosis by triggering the transition from Complex IIa to Complex IIb [1, 2, 33–35]. However, our findings indicate that the recruitment of CASP8 to RIPK1 may not exclusively rely on FADD. This conclusion is bolstered by the observation that the co-precipitation of FADD and RIPK1 within Complex II remains unaltered by the introduction of the S213E mutation in TNF or CASP8 precipitations (Figs. 2E and 4B). To further explore the recruitment of CASP8 to RIPK1, an inactivating mutant form of CASP8 (C360A) was co-expressed with both wild-type and mutant forms of RIPK1 in HEK293T cells. The results showed that wild-type RIPK1, as well as the D138N and S213A mutant forms, could directly bind to the CASP8 (C360A) mutant. However, the S213E mutant disrupted the binding between RIPK1 and CASP8 (Fig. 4C). Tests were also conducted to ascertain the direct binding of RIPK1 to the additional core components of complex IIa, specifically FADD and CFLAR, within HEK293T cells. The results indicated that the binding affinity between the S213E mutant and FADD remained unaltered. However, a modest reduction in binding was noted between the S213E mutant and CFLAR when compared to wild-type RIPK1 (Supplementary Fig. 4A, B). Subsequent analysis pinpointed the specific binding region of RIPK1 to CASP8, revealing that CASP8 engages its DED domains for binding, with RIPK1’s Death domain playing a prominent role in this interaction (Fig. 4D, E). Furthermore, these findings imply that the inhibition of RIPK1-dependent apoptosis observed in S213E cells may be attributed to disruptions not only in RIPK1 homodimerization but also in its interaction with CASP8.

## Discussion

The S213E mutation was initially detected through a functional screen targeting potential phosphorylation sites. However, despite our efforts, mass spectrometry has failed to detect a phosphorylation signal at the S213 site of RIPK1. Consequently, the phosphorylation status of the S213 site remains unconfirmed. Unlike other inhibitory mutations in the RIPK1 kinase domain, such as D138N, S25E, and T189E, which directly impair kinase activity [18–20, 36]. It was discovered that the S213E mutant lost its capacity for autophosphorylation at the S166 site. Previous research has indicated that the phosphorylation of S166 serves to enhance RIPK1 kinase activity and promote the formation of downstream death complexes [24]. However, in contrast to the S166A mutation, which only partially inhibits LZ-induced necroptosis, the S213E mutant exhibits complete inhibition of LZ-induced necroptosis. Importantly, S213E retains the ability to phosphorylate D138N at S166 (Fig. 2B), demonstrating preserved kinase activity. However, we observed a striking asymmetry in phosphorylation patterns: although D138N serves as a substrate for both S213E and wild-type RIPK1, wild-type RIPK1 rarely phosphorylates S213E (Fig. 2B, C). This suggests that the kinase domains of S213E and D138N adopt different conformations. The formation of RIPK1 homo-dimers via the death and RHIM domains, as well as the binding of RIPK3 through RHIM interaction, was disrupted by S213E. This indicates that the death and RHIM domains are shielded in S213E proteins. Therefore, we hypothesize that S213E confers a super-autoinhibitory state of RIPK1, preventing the recruitment of downstream effectors essential for RIPK1 function.

## Materials and methods

### Antibodies and reagents

The following antibodies and reagents were used in this study: RIPK1 (3493), P-S166-hRIPK1 (44590), P-S166-mRIPK1 (31122), hFADD (2782), hRIPK3 (13526), hCASP8 (9746), FLIP (56343), GAPDH HRP (8884), hCIAP2 (3130), NF-κB p65 (8242), P-S536-NF-κB p65 (3033), IκBα (4814), P-S32-IκBα (2859), Erk1/2 (4695), P-T202/Y204-Erk1/2 (4370), JNK (9252), P-T183/Y185-JNK (4668), p38 MAPK (8690), P-T180/Y182-p38 MAPK (4511) were purchased from Cell Signaling Technology. Pro CASP8 (ab108333), hMLKL (ab184718), P-S358-hMLKL (ab187091), P-S227-hRIPK3 (ab209384) were purchased from Abcam. Anti-Flag (F3165), Anti-Mouse IgG HRP (12-349), Anti-Rabbit IgG HRP (12-348) were purchased from Sigma. Anti-Myc (9E10) HRP (sc-40 HRP) was purchased from Santa Cruz. β-Actin HRP (PM053-7) was purchased from MBL. RIPK1 (610458) was purchased from BD. CL-CASP3 was purchased from R&D. Flag-mTNFα, hTNFα, hIFN-γ, hTRAIL were self-made by our laboratory. Smac mimetic (HY-15989), zVAD.fmk (HY-16658), Poly I:C (HY-107202), TAKi (HY-103490), TPCA-1 (HY-10074), NEC-1 (HY-15760), NSA (HY-100573), AP20187 (HY-13992) were purchased from MCE. CL-hPARP1 antibody was the gift from Dr. Gaofeng Fan of Shanghaitech University, Shanghai.

### Cell culture

HEK293T and HT-29 cells were cultured in DMEM (HyClone) supplemented with 10% FBS (Gemini) and 100 units/ml streptomycin/penicillin (HyClone). Cells were cultured at 37 °C with 5% CO2.

### Cell viability assay

Cell viability was determined by measuring the ATP luminescence using CellTiter-Glo Luminescent Cell Viability Assay (Promega) according to the manufacturer’s instructions. Luminescence was recorded with an EnSpire Multimode Plate Reader from PerkinElmer.

### Plasmids and molecular cloning

Full-length cDNAs for human/mouse RIPK1, RIPK3, CASP8, CFLAR, FADD and TRADD were PCR-amplified from the reverse-transcribed cDNA library. Mutant hRIPK1 and hCASP8 were generated using Gibson Assembly Cloning kit (NEB). Full-length, mutated or truncated cDNAs were cloned into the lentiviral vector pCDH-RFP/GFP (Addgene) with appropriate tags. For protein purification, hRIPK1 (1–294aa, WT or mutant) were cloned into BamHI/XhoI sites in pGEX-6p-1 plasmid. All plasmids were verified by DNA sequencing and the details of the plasmid sequences are available upon request.

### CRISPR/Cas9 knockout

HT-29 *RIPK1*-KO cells were generated by CRISPR/Cas9. The sgRNAs were cloned into the PX330 vector (Addgene). HT-29 cells were transfected with sgRNA plasmids using Lipofectamine 2000 (Invitrogen) or Lipofectamine 3000 (Invitrogen). Knockout cells were then selected by flow cytometry. The sgRNA sequences are listed below:

Human RIPK1: 5’-GCTCGGGCGCCATGTAGTAG-3’.

### Reconstitution of *RIPK1*-KO cells

For viral packaging, HEK293T cells were transfected with lentiviral vectors and virus packing plasmids psPAX2, pMD2.g (Addgene) using EZ-trans (Life-iLab) or PEI 40 K (MKbio). The medium was changed after 6 h, and collected at 48 h post-transfection. The virus-containing supernatant was then used to infect HT-29 cells with 10 μg/ml polybrene. Then viral infected cells were selected by flow cytometry and verified.

### Immunoblots and immunoprecipitation

For immunoblots, cells were lysed in lysis buffer containing 20 mM Tris-HCl, 150 mM NaCl, 1.5% Triton-X-100, 1 mM EDTA, 1 mM EGTA, 2 mM sodium pyrophosphate, 25 mM β-glycerophosphate, 10 mM NaF, 1 mM Na_3_VO_4_ and 10% glycerol (PH = 7.4). 1x Complete protease inhibitor cocktail (Roche) was freshly added to the lysis buffer prior to use. The protein concentrations of collected supernatants were determined by the Pierce BCA Protein Assay Kit (ThermoFisher). Lysates were then diluted in 4x loading buffer at 95°C for 10 min and then analyzed by SDS-PAGE.

For immunoprecipitation of complex I or complex II, cells were lysed in lysis buffer and the supernatants were immunoprecipitated overnight with anti-Flag beads (Biomake) at 4 °C, or incubated with anti-hCASP8 antibody (Cell Signaling Technology) overnight at 4 °C and then immunoprecipitated with Protein A/G agarose (Beyotime Biotechnology) for 4 h.

For Co-immunoprecipitation of overexpressed proteins in HEK293T cells, cells were lysed in lysis buffer after co-transfection for 24 h. The supernatants were immunoprecipitated overnight with anti-Flag beads (Biomake) at 4 °C. All immunocomplexes were eluted with 1× loading buffer at 95 °C for 10 min and then analyzed by SDS-PAGE.

### Protein expression and purification

Recombinant GST-hRIPK1 (1–294aa) proteins were expressed in BL21 (DE3) *Escherichia* *coli* after induction with 0.5 mM IPTG overnight at 16 °C. Bacteria were harvested by centrifugation and cell pellet was lysed in lysis buffer containing 25 mM Tris-HCl (PH = 7.4), 150 mM NaCl, 0.1 M PMSF (added fresh) with sonication or high-pressure homogenization at 4 °C for 15 min. GST-tagged protein was purified using Glutathione Sepharose 4B agarose (GE Healthcare) and eluted with 15 mM GSH. All the purified protein were concentrated and flash-frozen in liquid nitrogen and dried at −80 °C.

### In vitro kinase assay

For in vitro kinase assay, The GST tag of GST-hRIPK1 (1–294aa) was removed by overnight digestion with PreScission protease at 4 °C. 10 μg kinase-dead GST-hRIPK1 (1–294aa) and 2 μg hRIPK1 (1–294aa, WT and mutant) were mixed in 1× kinase reaction buffer containing 40 mM Tris-HCl (PH = 7.5), 20 mM MgCl_2_, 2 mM DTT, 100 μM ATP and 50 μM protease inhibitor. Kinase reactions were performed for 30 min at 30 °C and terminated by addition of 4× loading buffer and boiling at 95 °C for 10 min.

### Quantification and statistical analysis

All cell viability results were processed using GraphPad Prism 8. Results were shown as mean ± SD of at least three independent biological replicates. Group comparisons were performed using two-tailed Student’s *t*-test (two groups) or Two-way ANOVA (more than two groups). Differences were considered statistically significant if *p < 0.05; **p < 0.01; ***p < 0.001.

## Supplementary information

Supplementary Figure

Original data

## Data Availability

The original full size western blots are shown in the Original Data WB File.
