# Supplementary material for: RIPK1 S213E mutant suppresses RIPK1-dependent cell death by preventing interactions with RIPK3 and CASP8
Source: Cell Death Discov. 2025 Jul 25;11:345. doi: 10.1038/s41420-025-02647-x (PMC12297241; doi:10.1038/s41420-025-02647-x)
Supplement: Supplementary file 1 — Supplementary Figure [file 41420_2025_2647_MOESM1_ESM.docx]

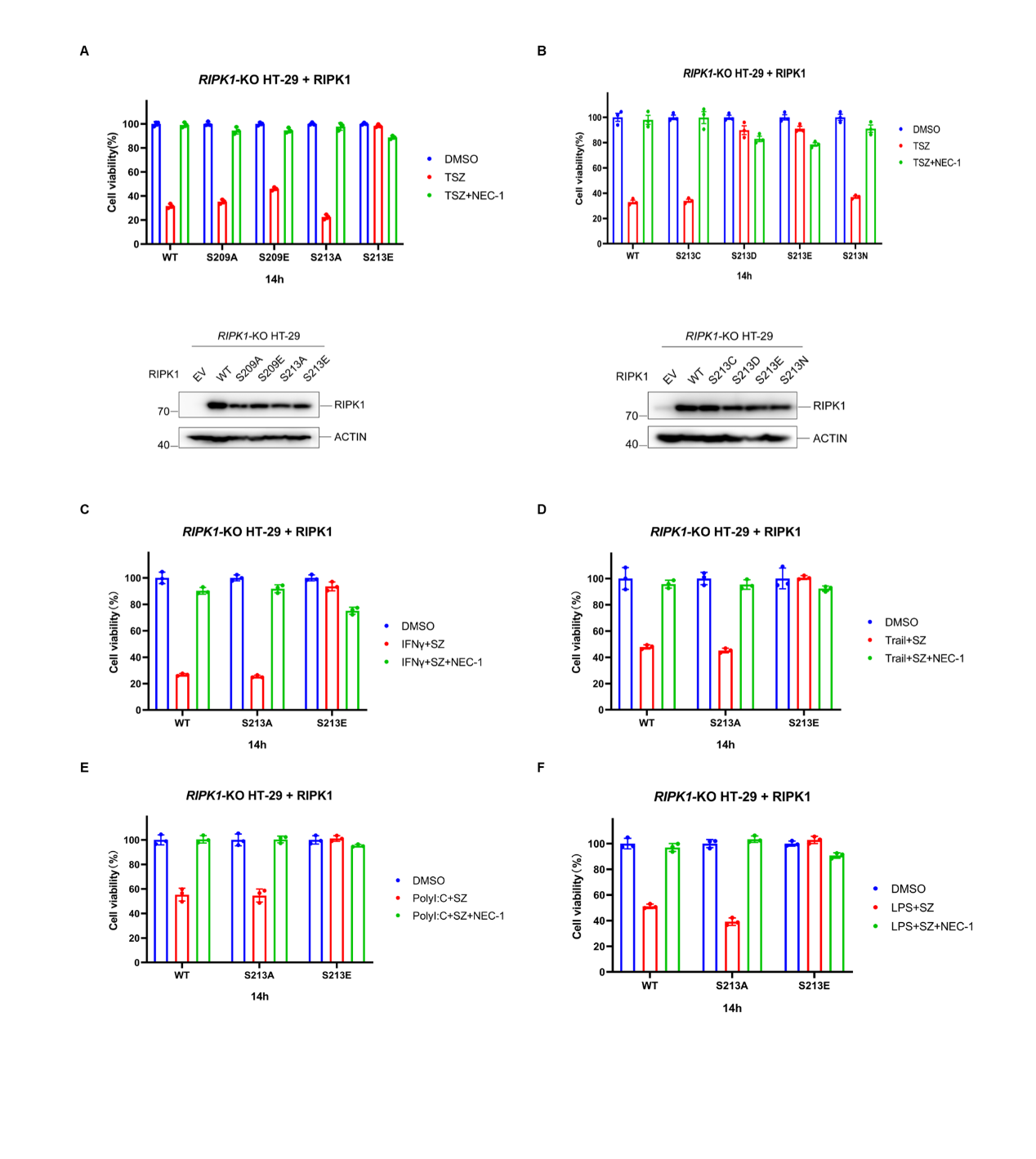


**Supplementary Fig. 1**  **S213E mutant blocks RIPK1 autophosphorylation and necroptosis. A** HT-29 *RIPK1*-KO cells were transfected with lentiviral vectors encoding wild-type or indicated RIPK1 mutants. Necroptosis was induced by treating with TSZ in the presence or absence of NEC-1 for 14 hours, cell viability was measured by CellTiter-Glo assay (**A. top**). The expression of RIPK1 mutants were determined by immunoblotting (**A. bottom**). **B** HT-29 cells of indicated RIPK1 wild-type or multiple S213 mutants were treated with TSZ in the presence or absence of NEC-1 for 12 hours, cell viability was measured by CellTiter-Glo assay (**B. top**). The expression of RIPK1 mutants were determined by immunoblotting (**B. bottom**). **C-F** HT-29 cells of indicated RIPK1 genotypes were treated with IFNγ (**C**), Trail (**D**), Poly I:C (**E**), LPS (**F**)+SZ in the presence or absence of NEC-1 for 14 hours, cell viability was measured by CellTiter-Glo assay. IFNγ: 100ng/ml; Trail: 40ng/ml; Poly I:C: 50μg/ml; LPS: 100ng/ml. The data are represented as the mean ± SD of *n*=3 independent experiments.


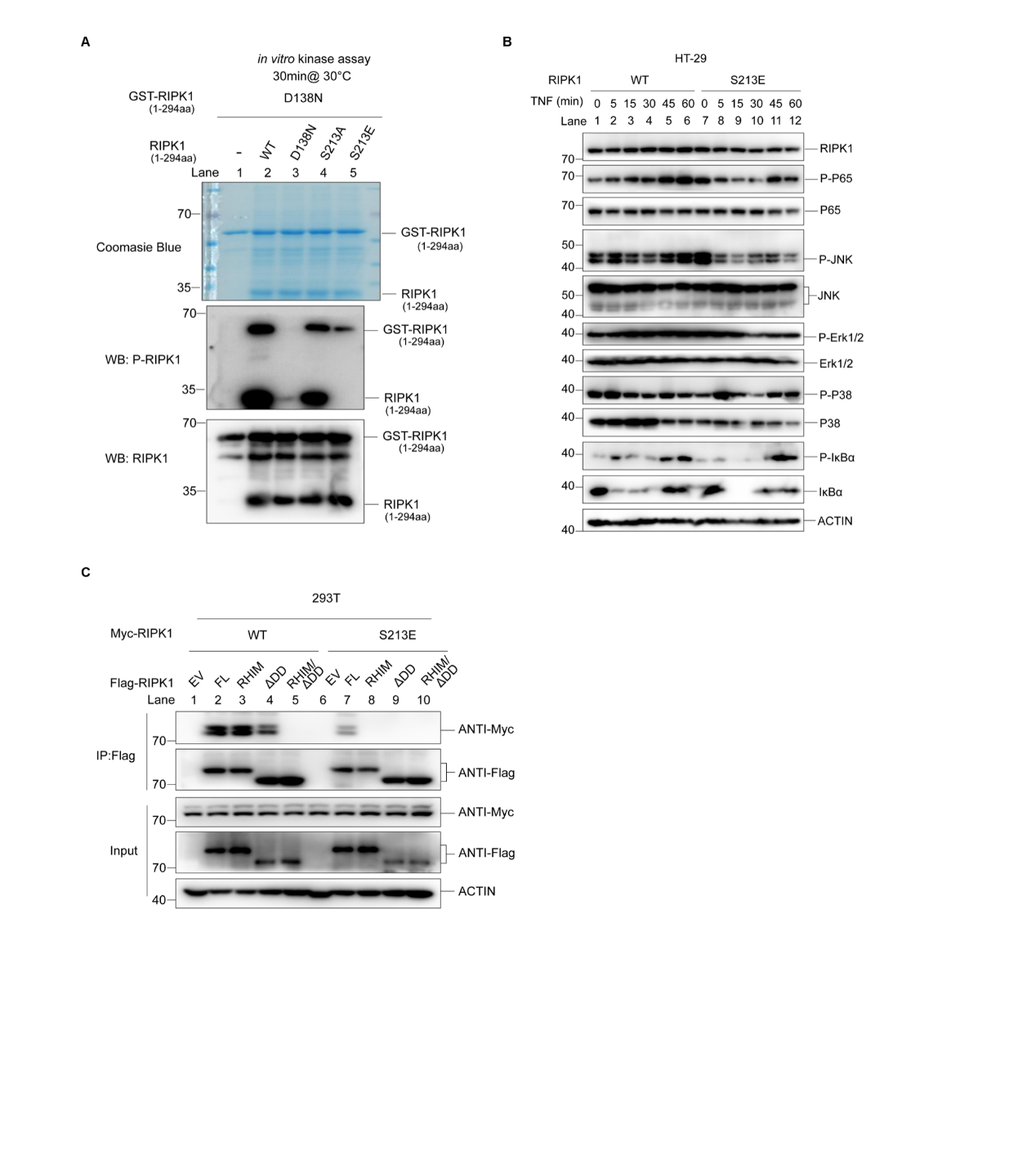


**Supplementary Fig. 2**  **S213E disrupts RIPK1 homodimerization and the interaction between RIPK1 and RIPK3. A** *In vitro* kinase assay of RIPK1 mutants. Recombinant GST-RIPK1 kinase domain (1-294aa) protein was purified from *E. coli*, GST tag was cleaved by ppase. GST-RIPK (1-294aa)-D138N (10μg) was incubated *in vitro* with or without RIPK1 (1-294aa)-WT, RIPK1 (1-294aa)-D138N, RIPK1 (1-294aa)-S213A, RIPK1 (1-294aa)-S213E (2μg) in the presence of 100μM ATP at 30℃ for 30min. The samples were separated by SDS-PAGE and stained with Coomassie blue. The levels of RIPK1 S166 autophosphorylation was determined by immunoblotting using anti-P-RIPK1 (S166) antibody. **B** HT-29 cells of RIPK1 WT and S213E were treated with TNF for indicated time points. The expression of IκBα, P-IκBα (S32), P-P65 (S536) and P-JNK (T183/Y185), P-Erk1/2 (T202/Y204), P-P38 MAPK (T180/Y182) were analyzed by immunoblotting using antibodies as indicated. **C** HEK293T cells were co-transfected with expression vectors for Flag-RIPK1-FL, Flag-RIPK1-RHIM, Flag-RIPK1-ΔDD, Flag-RIPK1-RHIM/ΔDD and Myc-RIPK1-WT, Myc-RIPK1-S213E as indicated for 24h. The cell lysates were immunoprecipitated using anti-Flag beads. The protein intereaction was analyzed by immunoblotting using antibodies as indicated. EV: empty vector; FL: full length; RHIM, RIPK1 RHIM motif core sequences “IQIG” mutant to “AAAA”; ΔDD, RIPK1 truncation without Death Domain.


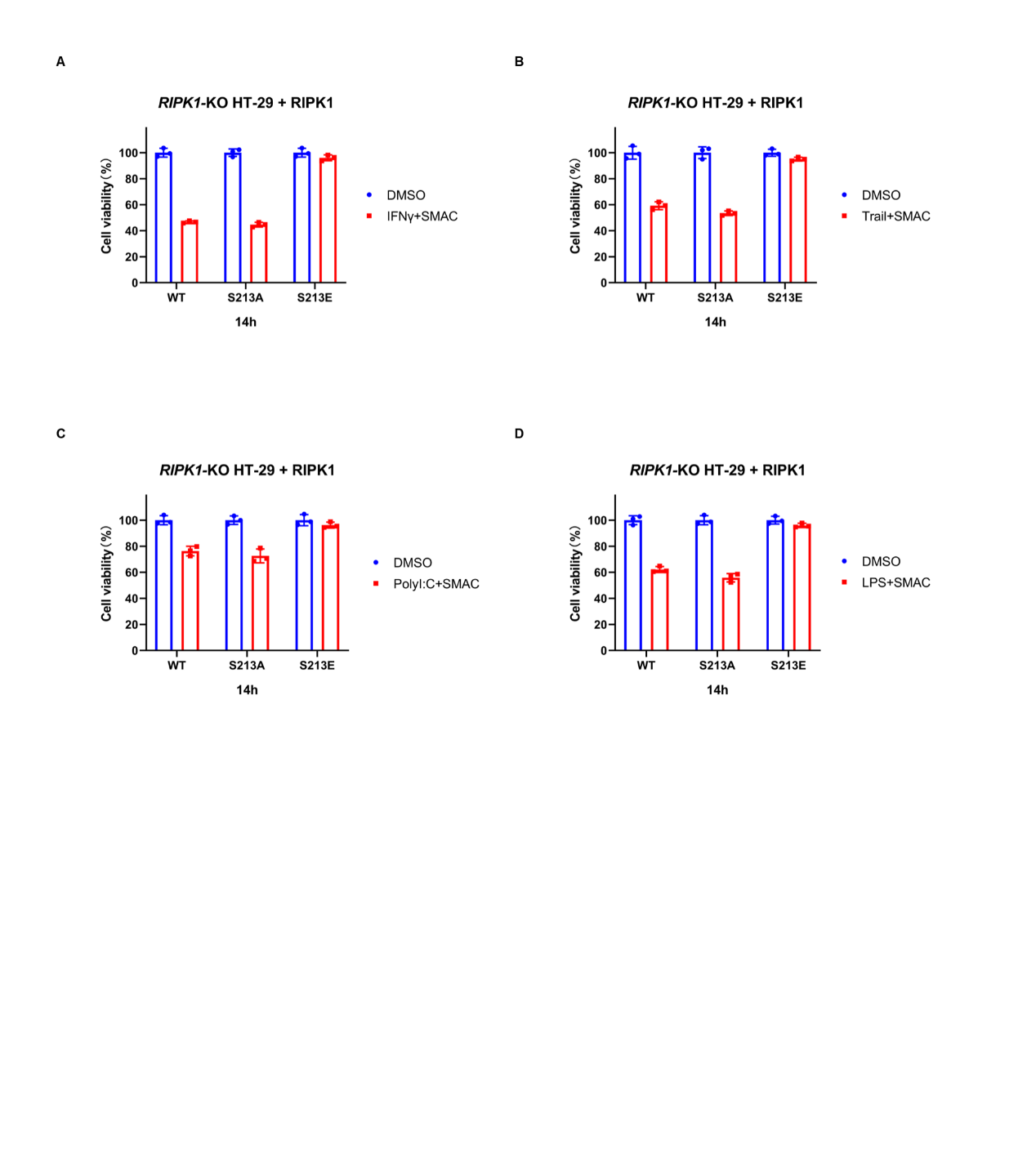


**Supplementary Fig. 3 S213E mutant blocks RIPK1-dependent apoptosis. A-D** HT-29 cells of indicated RIPK1 genotypes were treated with IFNγ (**A**), Trail (**B**), Poly I:C (**C**), LPS (**D**)+SMAC in the presence or absence of NEC-1 for 14 hours, cell viability was measured by CellTiter-Glo assay. IFNγ: 100ng/ml; Trail: 40ng/ml; Poly I:C: 50μg/ml; LPS: 100ng/ml. The data are represented as the mean ± SD of *n*=3 independent experiments.


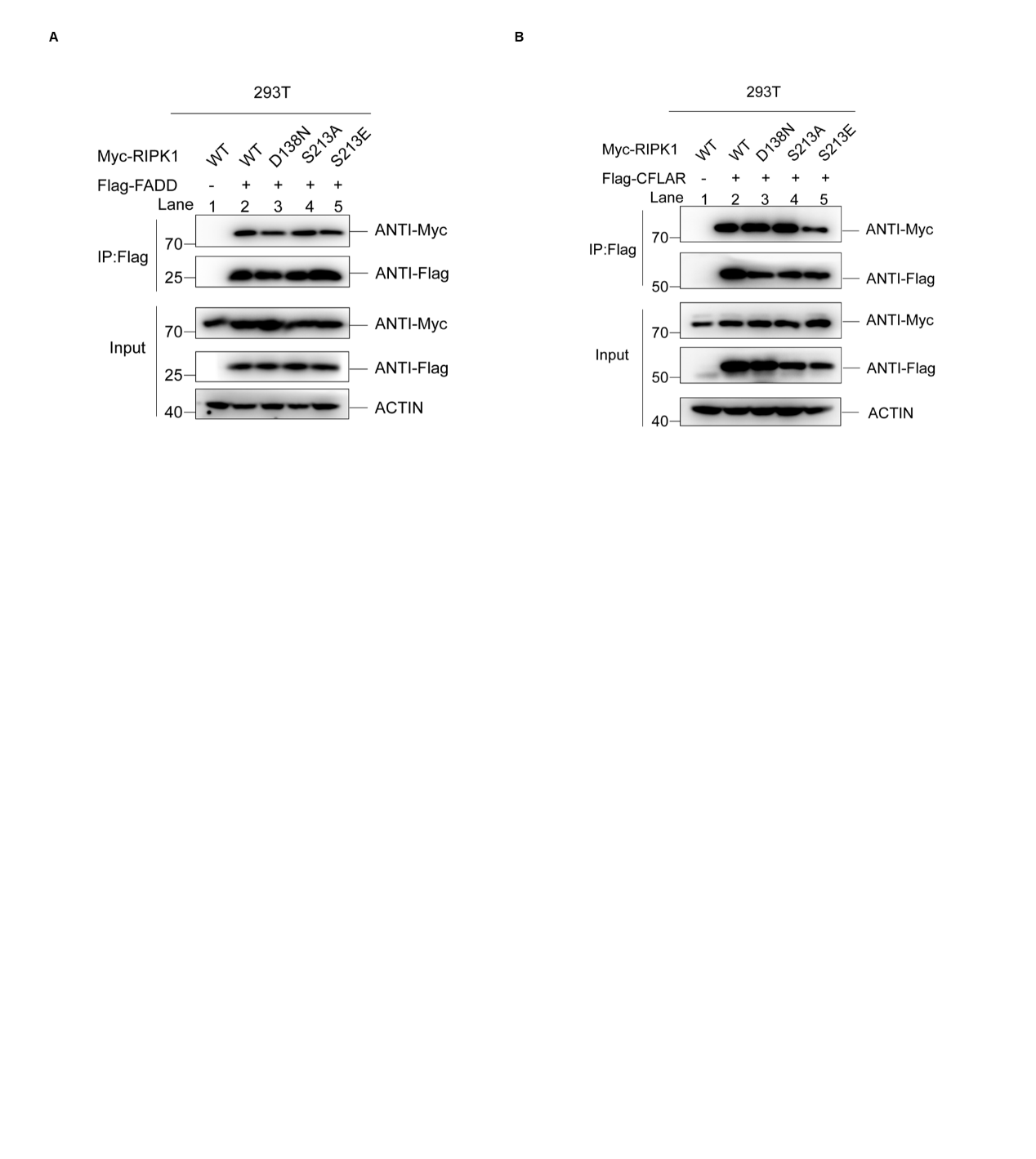


**Supplementary Fig. 4 S213E mutant does not disrupt the formation of Complex IIa, but the interaction of RIPK1 and CASP8. A-B** HEK293T cells were co-transfected with expression vectors for Myc-RIPK1-WT, Myc-RIPK1-D138N, Myc-RIPK1-S213A, Myc-RIPK1-S213E and Flag-FADD (**A**) or Flag-CFLAR (**B**) as indicated for 24h. The cell lysates were immunoprecipitated using anti-Flag beads. The protein intereaction was analyzed by immunoblotting using antibodies as indicated.
